# Dysregulation of platelet serotonin, 14–3–3, and GPIX in sudden infant death syndrome

**DOI:** 10.1038/s41598-024-61949-9

**Published:** 2024-05-15

**Authors:** Andrew L. Frelinger, Robin L. Haynes, Richard D. Goldstein, Michelle A. Berny-Lang, Anja J. Gerrits, Molly Riehs, Elisabeth A. Haas, Brankica Paunovic, Othon J. Mena, Steven C. Campman, Ginger L. Milne, Lynn A. Sleeper, Hannah C. Kinney, Alan D. Michelson

**Affiliations:** 1 Center for Platelet Research Studies, Dana-Farber/Boston Children’s Cancer and Blood Disorders Center, Harvard Medical School, Boston, MA USA; 2 Department of Pathology, Boston Children’s Hospital and Harvard Medical School, Boston, MA USA; 3 Robert’s Program on Sudden Unexpected Death in Pediatrics, Division of General Pediatrics, Department of Pediatrics, Boston Children’s Hospital and Harvard Medical School, Boston, USA; 4 Rady Children’s Hospital, San Diego, CA USA; 5 County of San Diego Medical Examiner’s Office, San Diego, CA USA; 6 County of Ventura Medical Examiner’s Office, Ventura, CA USA; 7 Division of Clinical Pharmacology, Vanderbilt University, Nashville, TN USA; 8 Department of Cardiology, Boston Children’s Hospital, Boston, MA USA; 9 Department of Pediatrics, Harvard Medical School, Boston, MA USA; 10 Center for Platelet Research Studies, Dana-Farber/Boston Children’s Cancer and Blood Disorders Center, Boston Children’s Hospital, Karp 08212, 300 Longwood Avenue, Boston, MA 02115-5737 USA

**Keywords:** Paediatric research, Diagnostic markers

## Abstract

Sudden infant death syndrome (SIDS) is the leading cause of post-neonatal infant mortality, but the underlying cause(s) are unclear. A subset of SIDS infants has abnormalities in the neurotransmitter, serotonin (5-hydroxytryptamine [5-HT]) and the adaptor molecule, 14–3–3 pathways in regions of the brain involved in gasping, response to hypoxia, and arousal. To evaluate our hypothesis that SIDS is, at least in part, a multi-organ dysregulation of 5-HT, we examined whether blood platelets, which have 5-HT and 14–3–3 signaling pathways similar to brain neurons, are abnormal in SIDS. We also studied platelet surface glycoprotein IX (GPIX), a cell adhesion receptor which is physically linked to 14–3–3. In infants dying of SIDS compared to infants dying of known causes, we found significantly higher intra-platelet 5-HT and 14–3–3 and lower platelet surface GPIX. Serum and plasma 5-HT were also elevated in SIDS compared to controls. The presence in SIDS of both platelet and brainstem 5-HT and 14–3–3 abnormalities suggests a global dysregulation of these pathways and the potential for platelets to be used as a model system to study 5-HT and 14–3–3 interactions in SIDS. Platelet and serum biomarkers may aid in the forensic determination of SIDS and have the potential to be predictive of SIDS risk in living infants.

## Introduction

Sudden infant death syndrome (SIDS) is defined as the sudden unexpected death of an apparently healthy infant less than one year of age that remains unexplained despite a complete autopsy with ancillary testing, examination of the death scene, and review of the clinical history^1,2^. SIDS is the leading cause of post-neonatal mortality in the United States^3–5^. Our laboratory has identified multiple abnormalities in SIDS, including brainstem (medullary) abnormalities in indices of serotonin (5-hydroxytryptamine, 5-HT) neurotransmission [e.g., 5-HT_1A_ receptor binding, 5-HT levels, tryptophan hydroxylase 2 (TPH-2) levels]^6–9^ and the 14–3–3 family of signaling proteins which have been shown to regulate many functions in brain development, including 5-HT synthesis^10,11^. In more recent work^12^, we identified significantly altered 5-HT_2A/C_ binding in SIDS cases in several key medullary nuclei overlapping with previously identified areas of reduced 5-HT_1A_ binding, suggesting abnormal signaling interactions between these 5-HT receptor subtypes in SIDS. Animal models that replicate the 5-HT anomalies present in SIDS cohorts have shown diminished survival responses (autoresuscitation) and death when challenged with an apneic event^13^. These findings inform our unified 5-HT brainstem hypothesis that posits a SIDS subset is due to 5-HT abnormalities in key nuclei of the rostral medullary 5-HT network that help mediate protective respiratory and autonomic responses to homeostatic challenges during sleep, autoresuscitation, and/or transitions to arousal^10,11^.

In addition to the brainstem 5-HT pathway abnormalities, we recently identified a significant increase in blood (serum) levels of 5-HT in SIDS cases compared to controls, raising the possibility of a global 5-HT dysregulation in SIDS^14^. The source of the elevated serum 5-HT in infants dying with SIDS remains unclear.

Peripheral blood platelets, made by megakaryocytes in the bone marrow, have been proposed as an easily accessible model of the neuronal 5-HT pathway because platelets, like neurons, have: (1) a 5-HT uptake mechanism^15^, (2) 5-HT stored in intracellular granules^16^, (3) activation-dependent release of 5-HT^17^, (4) the ability to respond to 5-HT via serotonergic receptors in the plasma membrane^18^, and (5) a mitochondrial enzyme, monoamine oxidase (MAO)^19,20^, which metabolizes 5-HT. Whereas serotonergic neurons possess TPH2 and are able to synthesize 5-HT^21^, platelets do not. Instead, circulating platelets take up 5-HT made by gut enterochromaffin cells^22^ and pulmonary neuroendocrine cells^23^ via the 5-HT transporter (SERT), and 5-HT is subsequently sequestered in platelet dense granules by the action of the vesicular monoamine transporter (VMAT)^24^. As a result, ~ 95% of the 5-HT in blood is sequestered within platelet dense granules^25^, where it is protected from the action of mitochondrial monoamine oxidases. While activation-dependent release of 5-HT from neurons is difficult to measure in living subjects, activation-dependent release of 5-HT from platelets can be estimated based on plasma 5-HT or metabolites, release of histamine, which is also stored in platelet dense granules and released upon activation^26^, or exposure of platelet dense granule membrane markers such as CD63^27^.

Platelets also contain 14–3–3 family members^28^ (ζ, β, γ, ε, η, and θ, but not σ) identical to the 14–3–3 molecules which regulate 5-HT synthesis in brains and are deficient in brains from SIDS cases compared to controls^10^. One function of 14–3–3 family members in platelets is to modulate platelet binding to von Willebrand factor and platelet adhesion via binding of 14–3–3 to the cytoplasmic tail of glycoprotein (GP) Ib, a part of the GPIb-IX-V cell adhesion receptor^28–30^. All 6 of the 14–3–3 family members expressed in platelets can bind GPIb-IX^30^.

Platelet biomarkers have not been studied in SIDS, but have been studied in some other neuronal disorders such as autism spectrum disorder (ASD)^31^ and epilepsy^32,33^. Platelet 5-HT levels are higher in ASD patients than controls and are associated with common platelet serotonin transporter (*SLC6A4*) and integrin beta3 (*ITGB3)* haplotypes^34,35^. In epilepsy, platelet serotonin transporter density is reduced following seizures^32,33^ and platelets themselves may promote seizures and contribute to neuroinflammation by modulating brain 5-HT^36^. Identification of platelet abnormalities in SIDS would provide new avenues for research and potential diagnostic tools for SIDS.

Thus, to test the hypothesis that our previously identified 5-HT pathway abnormalities in cardiorespiratory and arousal circuits of the brain in SIDS^10,11^ reflect a more global defect in the 5-HT pathway, in the present study we examined whether platelet 5-HT pathway and/or platelet 14–3–3 pathway biomarkers are also dysregulated in SIDS.

## Results

### Demographics of SIDS and non-SIDS cases

Post-mortem blood was collected from a cohort of SIDS (n = 50) and non-SIDS (n = 13) infants (Table 1). The causes of death of the controls were asphyxia (n = 4), accidental drowning (n = 1), cardiomyopathy (n = 1), pulmonary hypertension with cardiac defect (n = 1), subarachnoid hemorrhage due to cerebral vascular malformation (n = 1), meningoencephalitis (n = 1), moyamoya disease (n = 1), intestinal atresia (n = 1), acute nifedipine toxicity (n = 1) and opioid ingestion (n = 1). Cases and controls were not significantly different with respect to gender, gestational age at birth (~ 37 weeks, p = 0.533), or postmortem interval (~ 22.3 h, Table 1), but did significantly differ in post-conceptional age at death, postnatal age at death, death related to sleep, and position found (p < 0.01 for all, Table 1). Intrathoracic petechiae occurred in ~ 50% of both SIDS and non-SIDS cases (p = 1.00, Table 1). Although cases were accrued in a blinded fashion from October 2013 through January 2019, after adjudication it was noted that non-SIDS cases were more frequent in the period after July 2015 (Table 1).

**Table 1:** Subject characteristics.

Characteristic	SIDS	Controls	p
Number of cases	50	13	
Male, n (%)	33 (66.0%)	8 (61.5%)	0.755
Age in weeks, mean ± SD			
Postconceptional age at death	55.5 ± 11.8	69.4 ± 15.3	**0.001**
Gestational age at birth	37.9 ± 3.5	36.8 ± 5.3	0.533
Postnatal age at death	17.6 ± 12.2	31.7 ± 14.8	** < 0.001**
Postmortem interval in hours, mean ± SD	22.4 ± 8.1	22.2 ± 6.4	0.943
Collection date ≥ 07/25/2015, n (%)	29 (58.0%)	13 (100%)	**0.003**
Death related to sleep, n (%)			**0.004**
No	1 (2.0%)	4 (33.3%)	
Yes	49 (98.0%)	8 (66.7%)	
Position found, n (%)			**0.009**
Prone	19 (40.4%)	3 (27.3%)	
Supine	19 (40.4%)	2 (18.2%)	
Side	4 (8.5%)	0 (0%)	
Other	5 (10.6%)	6 (54.5%)	
Thoracic Petechiae, n (%)	25 (50%)	7 (53.9%)	1.00

Significant values are in bold.

### Serum and plasma 5-HT, 5-HT precursors and metabolites, and intra-platelet 5-HT

**Figure 1:**
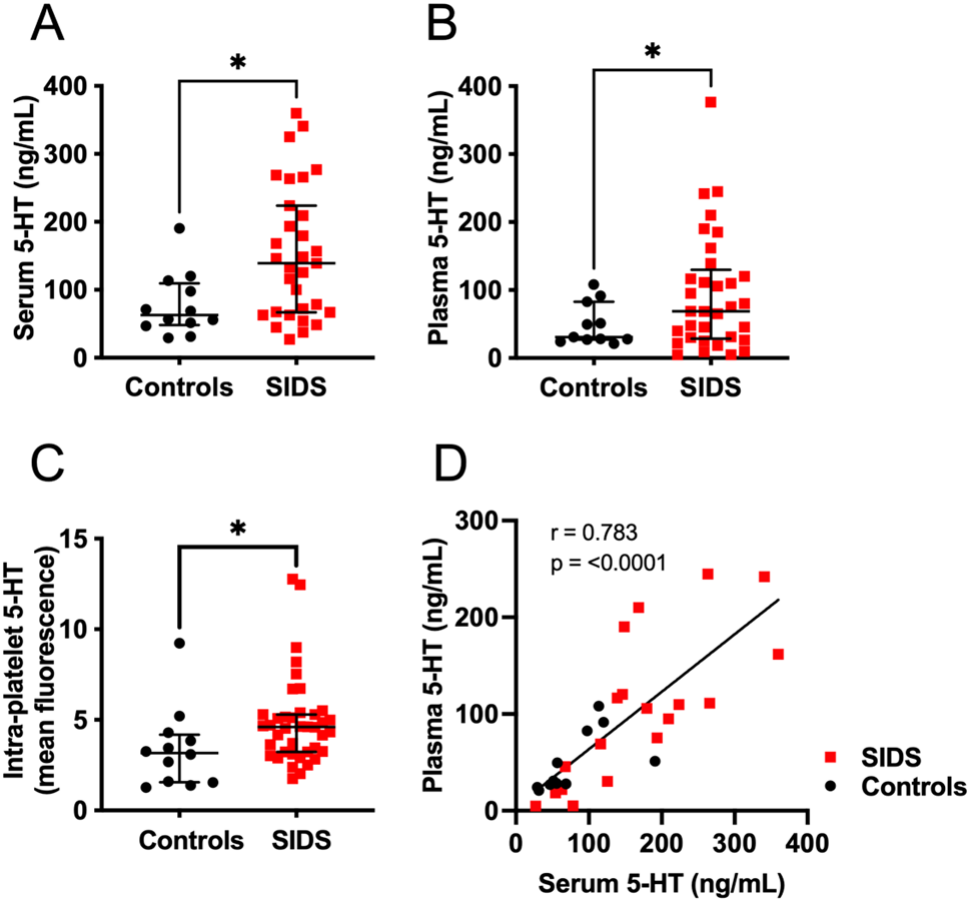
Serum, plasma and intra-platelet 5-HT in SIDS and control subjects. Lines indicate medians and interquartile ranges (IQR). Asterisks indicate p < 0.05 for ANCOVA SIDS vs. controls, with adjustment for collection date and post-conceptional age (**A**, **C**) or t test with Welch’s correction (**B**). (**D**) Pearson correlation of serum 5-HT and Plasma 5-HT in all subjects.

Platelet markers in SIDS cases were compared to those in control cases to rigorously account for possible unknown effects of post-mortem blood collection and sample processing on the measured parameters. Both serum and plasma levels of 5-HT were elevated in SIDS compared to controls (serum 5-HT 153.6 ± 97.0 ng/mL, adjusted mean ± SE vs. 77.8 ± 46.2 ng/mL, p = 0.012; plasma 5-HT 94.78 ± 85.64 vs. 49.32 ± 30.90 ng/mL, p = 0.013, Fig. 1A,B and Table 2). Tryptophan, the precursor of 5-HT, was not significantly different in serum of SIDS vs. control subjects (Table 2). Similarly, 5-HIAA, a metabolite of 5-HT, was not significantly different in serum or plasma of SIDS vs. control subjects (Table 2). Neither histamine (which, like 5-HT, is stored within platelet dense granules^25^) nor platelet surface CD63 (a marker of platelet dense granule release^37^) were significantly different in SIDS vs. controls (Tables 2 and 3).

**Table 2:** Serum and plasma 5-HT and related serum biomarkers.

Biomarker variable (ng/mL)	SIDS (n = 50)	Controls (n = 13)	t-test p-value
Serum 5-HT	153.63 ± 97.02	77.78 ± 46.16	0.012^a^
Serum tryptophan	13,101 ± 4,215	13,001 ± 4,412	0.946
Serum 5-hydroxyindoleacetic acid	41.20 ± 28.89	61.54 ± 40.35	0.268^a^
Serum histamine	44.05 (10.13)	39.53 (18.19)	0.844^a,b^
Plasma 5-HT	94.78 ± 85.64	49.32 ± 30.90	0.013^c^
Plasma 5-hydroxyindoleacetic acid	57.94 ± 24.71	78.75 ± 48.94	0.201^c^

LSmeans ± SD (or standard error).

^a^P value for log-transformed data.

^b^Adjusted for collection date.

^c^Welch’s correction (unpooled variance).

*5-HT* 5-hydroxytryptamine, *LSmeans* least squares means.

**Table 3:** Platelet biomarkers in SIDS and control cases.

Biomarker variable (geoMFI)	SIDS (n = 50)	Controls (n = 13)	p-value
Intra-platelet 5-HT	4.84 (0.39)	3.66 (0.77)	0.050^a,b^
Platelet surface CD63	18.05 (1.47)	23.53 (3.17)	0.097^a,b,c^
Intra-platelet 14–3–3ζ	36.71 (5.75)	64.08 (12.23)	0.010^a,b,c^
Platelet surface GPIX	242.38 (27.64)	526.64 (52.76)	< 0.001^a,c^
Platelet surface SERT	3.37 (0.252)	2.88 (0.490)	0.534^a,b,c^
Platelet surface 5-HT_2A_	4.27 (0.334)	3.51 (0.639)	0.306^a,b,c^
Total (permeabilized) platelet SERT	5.59 (0.54)	4.46 (1.08)	0.535^a,b,c^
Total (permeabilized) platelet 5-HT_2A_	29.24 (26.44)	29.86 (53.14)	0.675^a,b,c^

LSmeans (± standard error).

^a^P-value for log-transformed data.

^b^Adjusted for post-conceptional age.

^c^Adjusted for collection date.

*5-HT* 5-hydroxytryptamine, *geoMFI* geometric mean fluorescence intensity, *LSmeans* least squares means, *SERT* serotonin transporter.

Intra-platelet 5-HT was significantly higher in SIDS vs. controls (mean fluorescence intensity [MFI] 4.84 ± 0.39, mean ± standard error, vs. 3.66 ± 0.77, p = 0.050) (Fig. 1C and Table 3). Serum 5-HT was significantly correlated with plasma 5-HT (Fig. 1D, r = 0.783, p < 0.001), but not with intra-platelet 5-HT (r = 0.241, p = 0.163).

### Intra-platelet 14–3–3ζ

**Figure 2:**
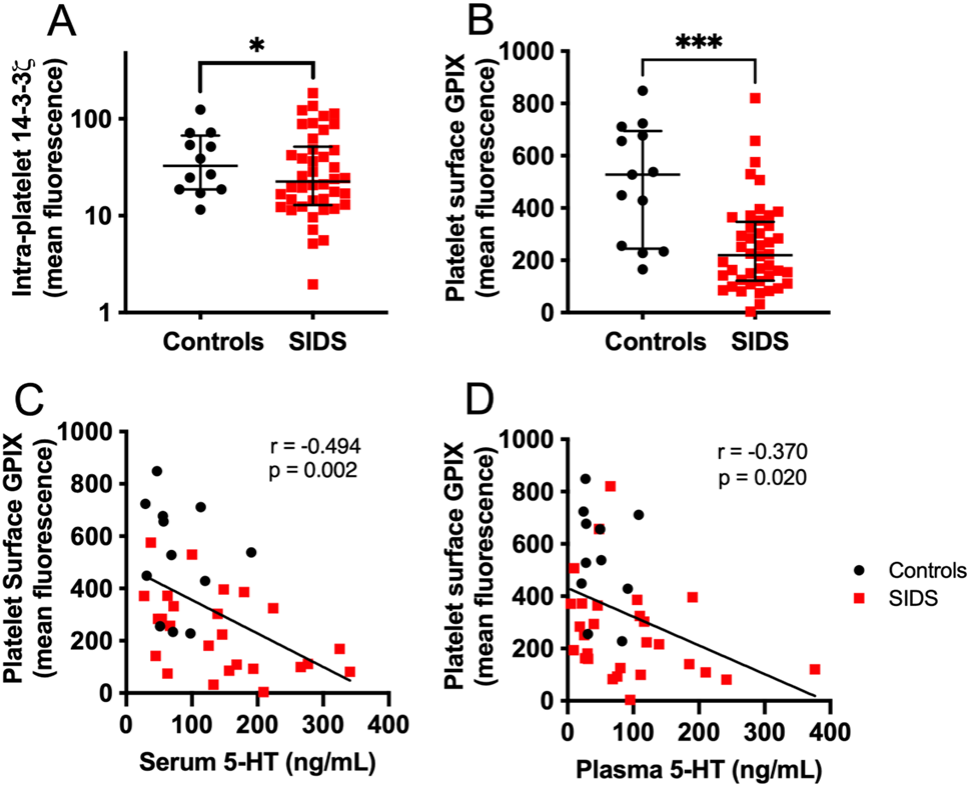
Platelet 14–3–3ζ and platelet surface GPIX in platelets of SIDS and control subjects. (**A**, **B**) Lines indicate median geometric mean fluorescence and IQR. Asterisks (*p < 0.05; ***p < 0.001) are for ANCOVA SIDS vs. controls, with adjustment for collection date and post-conceptional age. (**C**) Two-tailed Pearson correlation of serum 5-HT vs. platelet surface GPIX (all subjects) and (**D**) correlation of plasma 5-HT vs. platelet surface GPIX.

Platelet 14–3–3**ζ** MFI was significantly reduced in SIDS cases compared to controls (36.71 ± 5.75 vs. 64.08 ± 12.23, least square (ls) means ± SE with adjustment for post-conceptional age and collection date, p = 0.010, Fig. 2A and Table 3). Intra-platelet 14–3–3**ζ** was not significantly correlated with serum 5-HT (r = − 0.020, p = 0.911).

### Platelet surface GPIX

Platelet surface GPIX was significantly lower in SIDS cases (MFI 242.4 ± 27.6, ls mean ± SE) compared to controls (526.6 ± 52.8) (p < 0.001, Fig. 2B). Unlike intra-platelet 5-HT and 14–3–3**ζ** which were not correlated to serum 5-HT levels, platelet surface GPIX in SIDS and control samples was inversely correlated with serum 5-HT levels (Fig. 2C, r = − 0.494, p = 0.002). Likewise, platelet surface GPIX in SIDS and controls combined had a modest negative significant correlation with plasma 5-HT levels (Fig. 2D, r = − 0.370, p = 0.020).

### Platelet SERT and 5-HT_2A_ antigen and function

Platelet expression of SERT and the 5-HT_2A_ receptor, with and without permeabilization to assess total and surface expression, were not significantly different in SIDS cases compared to controls (Supplemental Material, Fig. S1, Table 3). While intra-platelet 5-HT and 14–3–3**ζ** and platelet surface GPIX and CD63 were all detectable above background isotype control antibody staining in both SIDS and control platelets, SERT function (as measured by mepacrine uptake) and 5-HT_2A_ function (as measured by 5-HT-stimulated increase in F-actin and cytosolic free calcium) were absent from postmortem SIDS and control platelets, but were present in fresh platelets collected from living donors (Supplemental Material, Fig. S2, Table S3). These results demonstrate that the assays for these endpoints were capable of detecting a functional response had it been present and are consistent with loss of SERT and the 5-HT_2A_ receptor function prior to sample analysis. Thus, whether SIDS and controls differed with respect to SERT function and 5-HT_2A_ function at the time of demise remains unknown.

## Discussion

The goal of the present study was to determine whether the 5-HT pathway dysregulation seen in cardiorespiratory and arousal circuits in the brains of SIDS patients^10^ also exists in platelets, a readily accessible model for neuronal 5-HT-signaling. In SIDS compared to control cases, our results show: (1) increased levels of plasma and intra-platelet 5-HT, (2) decreased levels of platelet 14–3–3**ζ** protein, (3) decreased levels of platelet surface adhesion receptor GPIX, and (4) in this independent cohort, confirmation of our previous finding^14^ of elevated serum 5-HT. Moreover, a correlation was observed between platelet surface GPIX and levels of both serum and plasma 5-HT. Thus, the differences between SIDS and controls in both platelet and brainstem 5-HT and 14–3–3 biomarkers suggest a global dysregulation of these pathways in SIDS.

Direct study of SIDS is inherently difficult due to the infrequent and unexpected nature of the disease, regulatory issues, and logistical and methodological issues surrounding specimen collection and storage. In the present study, regulatory issues were addressed by means of a California law which identified research on sudden infant death syndrome to be in the public interest and allows samples collected at autopsy to be made available for research. Platelet analysis requires free-flowing, unclotted blood which, somewhat surprisingly, is found at autopsy either because coagulation has not occurred or because fibrinolysis has taken place following postmortem coagulation. Previous studies have reported that platelets in postmortem blood are largely unactivated^38^ and initially aggregate in response to ADP, collagen and epinephrine, but reactivity decreases over time, with all reactivity lost by 10 h postmortem^39,40^. Due to the equipment and expertise required for platelet biomarker analysis, in the present study the collected specimens were shipped overnight to Boston Children’s Hospital. Nevertheless, the present results demonstrate the feasibility of measuring these biomarkers and enable future studies of these markers regardless of where the specimen is collected.

Gut enterochromaffin cells, pulmonary neuroendocrine cells (PNEC), and neuroepithelial bodies (NEBs) synthesize and release 5-HT^22,23^, making them possible sources for the elevated intra-platelet, plasma and serum 5-HT levels in SIDS. Moreover, hyperplasia and hypertrophy of PNEC/NEB occur within the lungs of infants classified as SIDS^41^ and there is a novel population of PNECs (as defined by the marker TUBB3) in lungs of SIDS cases with high serum 5-HT compared to that in SIDS cases with normal serum 5-HT^42^.

To investigate whether decreased 5-HT metabolism contributes to increased plasma and serum 5-HT in SIDS, we measured serum tryptophan (the precursor for synthesis of 5-HT) and plasma and serum 5-HIAA (a 5-HT degradation product) but found no difference in these end points (Table 2). Since plasma 5-HT is sequestered by circulating platelets^22^, we examined platelet levels of 5-HT. Our finding of significantly elevated intra-platelet 5-HT in SIDS (Fig. 1C and Table 3) provides an explanation for the elevated serum 5-HT, and raised the possibility that platelets in SIDS may sequester more 5-HT than control cases. However, no difference in platelet surface and total platelet SERT was found (Fig. S1). Whether platelet SERT function is increased in SIDS and contributes to elevated intra-platelet 5-HT remains unclear since SERT function is lost in all postmortem samples (Fig. S2, Table S3).

Higher plasma and serum 5-HT in SIDS compared to controls would be consistent with increased platelet activation and corresponding dense granule release. Greater platelet activation in SIDS would also account for the presently observed reduced levels of platelet surface GPIX (Fig. 2), which, together with GPIb, is internalized following platelet activation^43^. However, serum levels of histamine, which is also stored in platelet dense granules, were not significantly different for SIDS compared to control cases (Table 2). While this would appear to argue against platelet activation as a cause of higher plasma and serum 5-HT in SIDS and the decrease in platelet surface GPIX in SIDS subjects, under some physiologic conditions the activation-dependent decrease in platelet surface GPIX does not correlate with platelet dense granule release (as reflected by increased platelet surface CD63 and histamine release)^44^ or platelet alpha granule release (as reflected by increased platelet surface P-selectin)^45^. Nevertheless, the increased plasma, serum and intra-platelet 5-HT in SIDS compared to controls provides clear evidence that 5-HT abnormalities in SIDS are not restricted to the brain^10^.

14–3–3 is an adaptor molecule which, among many other functions^46^, regulates 5-HT biosynthesis in neurons by modulating TPH-2 activity in neurons^47^ and modulates platelet adhesion and aggregation activity through interactions with GPIb-IX and GPIIb-IIIa in platelets^28,30,48^. Using one-dimensional sodium dodecyl sulfate–polyacrylamide gel electrophoresis followed by liquid chromatography-tandem mass spectrometry (GeLC-MS/MS) in frozen tissue, we previously reported a 42–75% reduction of 14–3–3 isoforms in the gigantocellularis of the medullary 5-HT system in SIDS compared to controls^10^. Six isoforms were identified by MS and 4 were significantly different: γ, ε, β, and θ. 14–3–3**ζ** was also reduced in SIDS (p = 0.068, which may be considered marginally significant considering the small sample size). By Western blot analysis, significant decreases were confirmed in γ, ε, and β. Our finding of decreased 14–3–3 family members differed from that of Hunt et al.^49^ using laser desorption/ionization imaging mass spectrometry (MALDI-IMS) in formalin fixed paraffin-embedded (FFPE) archival tissue. Hunt et al.^49^ did not find differences in any 14–3–3 family of proteins. The following technological differences likely account, at least in part, for the different findings among the studies: (1) differences in ionization techniques (MALDI vs electrospray (ESI); (2) differences in proteomic platforms (imaging vs bulk tissue); and (3) differences in tissue type (FFPE vs frozen). The 2 studies also differed in their overall protein identification. Hunt et al.^49^ identified 55 proteins based on 285 peptides while we identified ~ 250 proteins based on ~ 1000 peptides^10^. Given these technological and recovery differences, variations in the results are not unexpected. In the present study, intra-platelet 14–3–3**ζ** was significantly decreased in platelets from SIDS compared to control cases (Fig. 2A, Table 3). Pilot studies with antibodies to other 14–3–3 isoforms yielded insufficient or poorly reproducible staining of platelets and were therefore not included in the study. Because the decrease in intra-platelet 14–3–3**ζ** was not correlated with the increases in intra-platelet 5-HT, plasma 5-HT or serum 5-HT, it remains unclear whether these abnormalities are mechanistically related.

Reduced intra-platelet 14–3–3**ζ** levels in SIDS have the potential for effects across a range of platelet functions. Mice deficient in 14–3–3**ζ** show decreased platelet phosphatidylserine exposure and thrombin generation^50^. In addition, platelet 14–3–3**ζ**, via binding to the cytoplasmic domain of GPIbα, has been shown to regulate GPIb-IX binding to von Willebrand factor and GPIb-IX-mediated platelet adhesion^28–30^. Platelet 14–3–3**ζ** also contributes to platelet integrin (GPIIb-IIIa)-dependent outside-in signaling^29^, which initiates and amplifies platelet spreading, thrombus consolidation and clot contraction^51,52^. Taken together, these studies suggest that decreased intra-platelet 14–3–3**ζ** in SIDS may result in reduced platelet function and a hemorrhagic phenotype. Prior studies have reported more intrathoracic (thymus, lungs, pleura and epicardium) petechiae^53–56^ and liquid heart blood^57,58^ in SIDS compared to controls. However thoracic petechiae were equally frequent in SIDS compared to controls in the present cohort (Table 1).

Platelet surface GPIX, which was examined because of its physical link to 14–3–3^30^, was significantly reduced in SIDS compared to control cases (Fig. 2B and Table 3). Because the magnitude of the decrease in GPIX was much larger than the change seen in 14–3–3**ζ** (Table 3), it is unlikely that the changes in 14–3–3**ζ** solely account for the changes in GPIX. Rather, this novel and unexpectedly large difference in platelet surface GPIX in SIDS vs controls represents an opportunity for new areas of research in SIDS. Reduced platelet surface GPIX occurs in patients with Bernard-Soulier syndrome (BSS), but genetic studies of SIDS populations have not identified mutations in BSS-causing genes^59^. As mentioned above, down-regulation of platelet surface GPIX can also be caused by platelet activation^45^ which may be triggered or enhanced via multiple pathways, including infection and inflammation^60–62^, both of which have been suggested to be increased in SIDS^63^.

Recent studies suggest unrecognized infection and neuroinflammation may be present in a subset of SIDS^64^ and that platelets represent a link between the periphery and neuroinflammation^65–68^. In the setting of epilepsy, platelets themselves are reported to directly contribute to neuroinflammation^36^. Specifically, platelet degranulation near the blood brain barrier may directly affect the brain endothelium and factors released by platelets, including 5-HT, may alter neuronal activity^36^. Therefore, the presently reported platelet biomarker abnormalities raise the possibility that platelets in SIDS may also contribute to neuroinflammation.

A limitation of this study is the size of the control group (n = 13). However, small sample size for controls in SIDS studies reflect the real-world difficulty in collecting such specimens and is common in SIDS publications^8,49,69^. There were statistically significant differences between SIDS and controls in the age at death and date of collection (Table 1), but these differences were accounted for by ANCOVA analyses with adjustment for these covariates. Finally, although platelet 14–3–3**ζ** was significantly reduced in SIDS vs controls here (Fig. 2A), other 14–3–3 isoforms exist in platelets which were not measured due to insufficient antibody staining.

In conclusion, differences between SIDS and controls in both platelet and brainstem biomarkers of 5-HT and 14–3–3 pathways suggest a global dysregulation of these pathways in SIDS and demonstrate the feasibility of platelets as an easily accessible (compared to brain) model of neuronal changes in SIDS. Moreover, the reduced level of platelet surface GPIX in SIDS is a novel finding which opens new areas of investigation. For example, future studies of platelet surface GPIX in parents of infants dying of SIDS may provide insight on whether the reduced platelet surface GPIX in SIDS subjects is inherited. The present findings of abnormal platelet 5-HT and 14–3–3**ζ** in SIDS also suggest that further investigation of related markers such as platelet VMAT and MAO and plasma or serum levels of 14–3–3 family members is warranted. Future prospective studies are necessary to understand the potential of platelet, plasma and serum 5-HT, platelet 14–3–3**ζ**, and platelet GPIX to aid in the forensic determination of SIDS in autopsied infants. Forensic tests that distinguish SIDS cases from others on the basis of abnormal 5-HT, platelet 14–3–3**ζ** and/or platelet GPIX would provide comfort to grieving parents and help to remove a potentially devastating stigma. Because SIDS is, by definition, the sudden, unexplained death of an infant, specimens are not available to determine whether these biomarkers are altered in living infants who subsequently develop SIDS. Thus, whether serum, plasma or platelet 5-HT, or the other platelet biomarkers identified here, all of which are available in easily collected peripheral blood, identify living individuals at risk for SIDS requires focused studies in high risk populations or large, long-term, prospective population studies.

## Materials and methods

A cohort of SIDS and non-SIDS cases was accrued from October 2013 through January 2019 in collaboration with the San Diego Office of the Medical Examiner in San Diego County, CA. All methods were carried out in accordance with relevant guidelines and regulations. Specimens were available for research under the auspices of California Code, Section 27491.41 which authorizes that tissues from infants dying suddenly and unexpectedly under the jurisdiction of the medical examiner may be used for research without direct parental consent. All experimental protocols were approved by Boston Children’s Hospital Institutional Review Board. Case and control adjudications were performed blinded to laboratory results obtained in this research study and were based on autopsy reports, clinical information, and death scene investigations. All SIDS cases were sudden, unexpected deaths of infants under 1 year of age that remained unexplained after a complete autopsy and death scene investigation^1^. Controls were previously healthy infants who died of definable acute disorders with no or only minor signs of clinical illness within 1 week of death. Clinical variables, e.g., gestational age at birth, postnatal age at death, gender, race, perinatal and/or postnatal illnesses, and time of death were recorded. Variables related to the sleep environment, i.e., sleep position at discovery or history of bed-sharing the night of death, were also recorded, as well as variables related to acute illness 48 h and 1 week before death. Postmortem interval (PMI), the time between death and autopsy, was also recorded.

**Figure 3:**
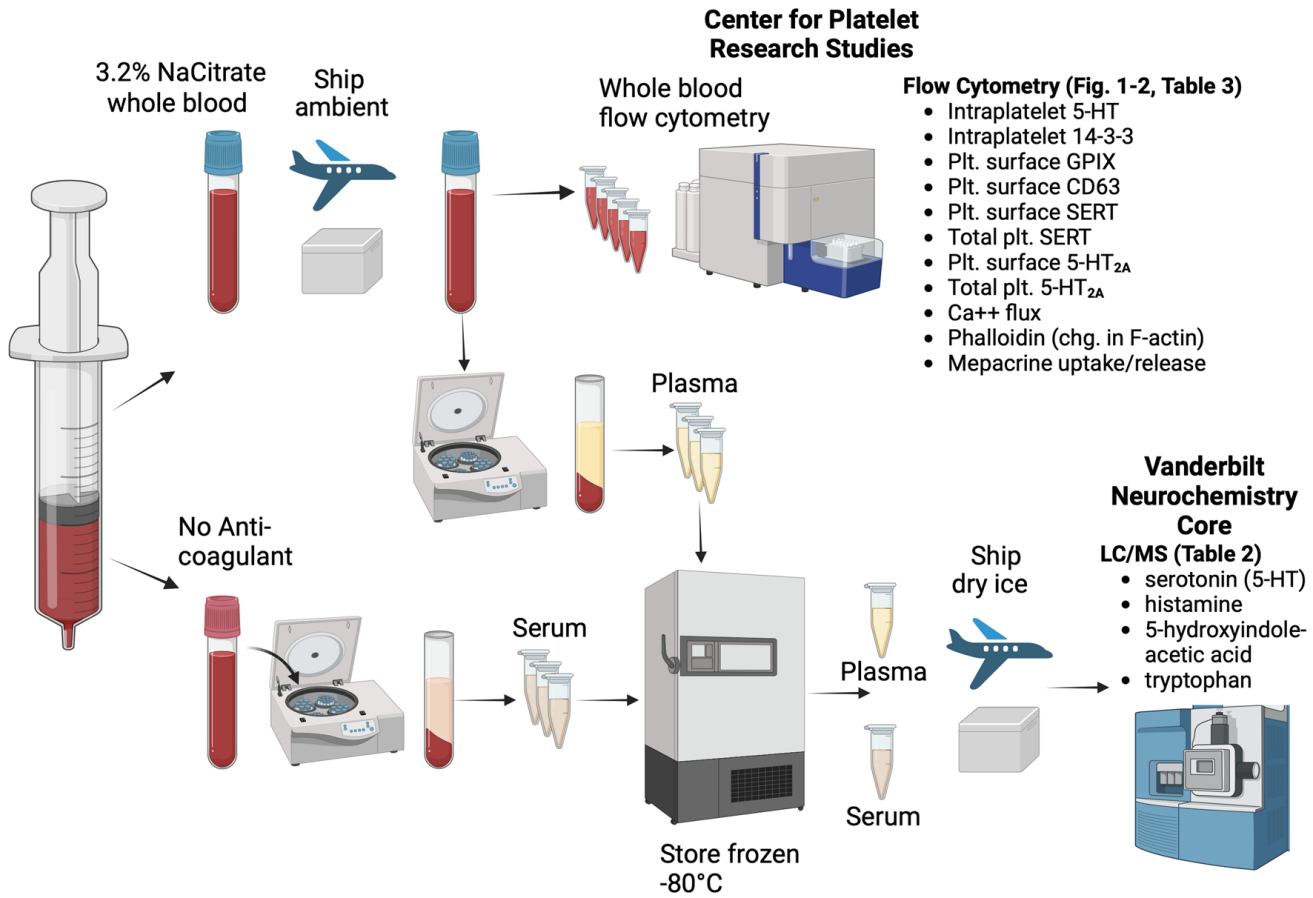
Overview of sample processing logistics and endpoints studied.

**Figure 4:**
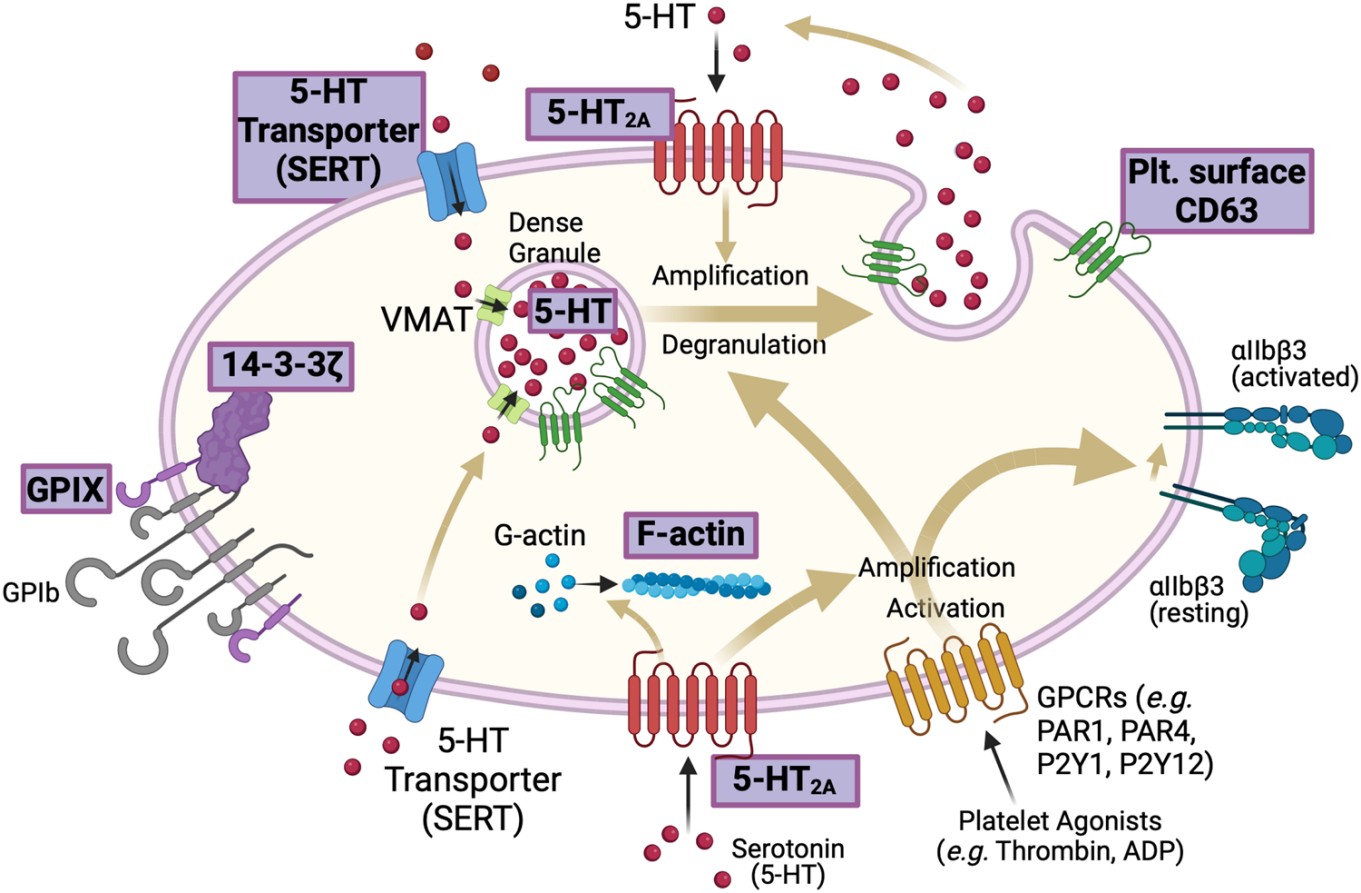
Schematic of platelet biomarkers measured by whole blood flow cytometry. Endpoints measured are highlighted in purple. Intracellular markers, 14–3–3ζ, 5-HT, and F-actin, were measured after fixation of platelets with formaldehyde and permeabilization with Triton-X 100. Created with BioRender.com.

Blood was collected at autopsy from large blood vessels into Vacutainer™ citrate (3.2%) tubes (BD Biosciences, San Diego, CA) for platelet studies and Vacutainer SST serum separator tubes (containing a silica clot activator and polymer gel) for serum separation and analysis of serum analytes. Citrate-anticoagulated whole blood samples were shipped overnight at ambient temperature to the Center for Platelet Research Studies (Boston Children’s Hospital) for analysis of platelet biomarkers. Platelet markers in SIDS cases were compared to those in control cases to rigorously account for possible unknown effects of post-mortem blood collection and sample processing on the measured parameters. As a quality control measure, all flow cytometric assays were intermittently evaluated against blood from live controls to ensure consistent reagent performance. The remainder of the citrated blood was centrifuged to prepare plasma. Serum and plasma were stored frozen at − 80 °C until analysis in ~ 0.5–1.0 mL aliquots. Samples were thawed once for aliquoting prior to MS/LC analysis. Figures 3 and 4 provide an overview of sample processing and endpoints.

### Plasma and serum 5-HT and 5-hydroxyindole acetic acid (5-HIAA) and serum tryptophan and histamine

Plasma and serum 5-HT and 5-HIAA and serum tryptophan and histamine were measured by mass spectrometry at the Vanderbilt Neurochemistry Core, Vanderbilt University School of Medicine following derivatization with benzoyl chloride, as previously described^70^.

### Internal standard synthesis

Stock solutions of 5-HT, 5-HIAA, and histamine (5 ng/µL each) were made in DI water and stored at − 80 °C. To prepare internal standards, stock solutions were derivatized in a similar manner to samples using isotopically labeled benzoyl chloride (^13^C_6_-BZC) as follows: 200 µL of the stock solution was mixed with 400 µL each of 500 mM NaCO_3_ (aq) and 2% ^13^C_6_-BZC in acetonitrile was added to the solution. After 2 mins, the reaction was stopped by the addition of 400 µL 20% acetonitrile in water containing 3% sulfuric acid. The solution was mixed well and stored in 10 µL aliquots at − 80 °C. One aliquot was diluted 100 × with 20% acetonitrile in water containing 3% sulfuric acid to make the working internal standard solution used in the sample analysis.

### Benzoyl chloride derivatization and LC/MS analysis

Analytes in plasma were quantified using liquid chromatography/mass spectrometry (LC/MS) following derivatization with benzoyl chloride (BZC)^70^. 20 µL of blood was diluted with 60 µL acetonitrile:water (80:20), vortexed, and allowed to sit on ice for 10 min. The solution was then spun at 3.5 g for 5 min to pellet proteins. 5 µL of supernatant was then mixed with 10 µL each of 500 mM NaCO_3_ (aq) and 2% BZC in acetonitrile in an LC/MS vial. After 2 min, the reaction was stopped by the addition of 10 µL internal standard solution.

LC was performed on a 2.1 × 100 mm, 1.6 µm particle CORTECS Phenyl column (Waters Corporation, Milford, MA, USA) using a Waters Acquity UPLC. Mobile phase A was 0.1% aqueous formic acid and mobile phase B was acetonitrile with 0.1% formic acid. MS analysis was performed using a Waters Xevo TQ-XS triple quadrupole tandem mass spectrometer. The source temperature was 150 °C, and the desolvation temperature was 400 °C. The LC gradient and MS settings for 5-HT and 5-HIAA are shown in Supplemental Tables S1 and S2.

### Platelet surface GPIX

Platelet surface GPIX was measured by whole blood flow cytometry as previously described^43,45,71–73^. Briefly, citrate-anticoagulated whole blood (10 µL) was incubated 15 min RT with an antibody cocktail consisting of CD42a-PE (phycoerythrin, to detect GPIX, catalog # 558819, BD Pharmingen, San Diego, CA), CD42b-FITC (fluorescein isothiocyanate, catalog # 555472, BD Pharmingen) and CD41-PerCP-Cy5.5 (peridinin chlorophyll protein-Cyanine5.5, catalog # 340930, BD Biosciences, San Diego, CA) in the presence of vehicle (10 mM HEPES, 0.15 M NaCl, pH 7.4, “HEPES-saline”), adenosine diphosphate (ADP, catalog # 384, ChronoLog, Havertown, PA) 20 µM, or thrombin receptor activating peptide (TRAP, catalog # 4031274, BaChem, Torrance, CA) 20 µM (total volume 25 µL). Samples were then fixed by addition of 1% formaldehyde in HEPES-saline. Control samples with FITC and PE conjugated normal IgG were used to establish levels of non-specific staining. Samples were analyzed in a FACS Calibur flow cytometer (BD Biosciences, San Jose, CA) (threshold on FL3 CD41-PerCP-Cy5.5) and gated for platelet forward and side light scatter. Compensation for fluorescence overlap of the channels was determined using single stained samples.

### Intra-platelet 14–3–3ζ

Rabbit polyclonal antibody to 14–3-3**ζ** (cat# ab63635) was purchased from Abcam (Cambridge, MA). Diluted (1:4) whole blood was fixed with formaldehyde then incubated 30 min at RT with rabbit anti-14–3–** ζ** and CD41-PerCP-Cy5.5 (catalog # 340930, BD Biosciences) in 0.2% Triton X-100, followed by 30 min incubation with goat anti-rabbit Dylight 488 (Abcam, Cambridge, MA). Samples were then diluted with 500 µL HEPES-saline buffer and immediately analyzed by flow cytometry. Control samples containing non-specific rabbit IgG instead of anti-14–3–3 were used to establish levels of non-specific staining.

### Intra-platelet 5-HT

Citrate-anticoagulated blood was diluted fivefold in HEPES-Tyrode’s buffer then mixed with an equal volume of 2% formaldehyde in HEPES-saline for 30 min. Diluted fixed blood was incubated 30 min at RT with FITC-conjugated rabbit polyclonal anti-5-HT (catalog # orb16705, Biorybt, Durham, NC) and CD41a-PerCP-Cy5.5 in the presence of 0.2% Triton X-100. Staining was stopped by addition of 1% formaldehyde in 10 mM HEPES, 0.15M sodium chloride, pH 7.4, and samples were analyzed by flow cytometry.

### Platelet surface and total 5-HT_2A_ and SERT antigen

Platelet levels of 5-HT_2A_ and SERT were evaluated by flow cytometry on fixed platelets without permeabilization (to assess platelet surface expression) and with permeabilization (to assess surface plus intracellular platelet expression). Citrate-anticoagulated blood was diluted fivefold in HEPES-Tyrode’s buffer (10 mM HEPES, 137 mM sodium chloride, 2.8 mM potassium chloride, 1 mM magnesium chloride, 12 mM sodium hydrogen carbonate, 0.4 mM sodium phosphate dibasic, 5.5 mM glucose, and 0.35% w/v bovine serum albumin, pH 7.4), then mixed with an equal volume of 2% formaldehyde in HEPES-saline for 30 min. The fixed samples were then diluted ~ 1:4 with HEPES-saline to reduce the formaldehyde concentration prior to incubation of the samples with antibodies. Fixed diluted blood was mixed in parallel assays with FITC-conjugated rabbit polyclonal antibodies against 5HT_2A_ (catalog # orb16708) and SERT (catalog # orb13779) from BioRbyt (distributed by Biocompare, South San Francisco, CA) with and without addition of 0.2% Triton X-100 (final concentration). After 30 min, samples were fixed by addition of 1 mL 1% formaldehyde in HEPES-saline. Platelets were identified using CD41a-PerCP-Cy5.5.

### Platelet SERT function

The function of the platelet surface 5-HT transporter, SERT, was evaluated by assessing uptake of the fluorescent 5-HT analog quinacrine (mepacrine, Sigma, St. Louis, MO) as previously described^74^. Citrate-anticoagulated blood was diluted 1:35 in HEPES-Tyrode’s buffer and pre-labeled for 5 min at 37 °C with CD41a-PerCp-Cy5.5. Aliquots of pre-labeled samples (144 µL) were mixed at 37 °C with quinacrine (16 µL, 1 mM final concentration) or vehicle and at 1, 5, 10 and 30 min, 25 µL samples removed, diluted with 500 µL HEPES-saline, and analyzed immediately by flow cytometry.

### Platelet 5-HT_2A_ receptor function

Platelet 5-HT_2A_ receptor-mediated functions were assessed by measuring 5-HT-stimulated calcium flux and 5-HT-stimulated actin polymerization using methods previously described^75^. Briefly, 5-HT-stimulated F-actin polymerization was assessed by phalloidin binding. Blood was diluted 1:4 in HEPES-Tyrode’s buffer, stimulated for 25 s at 22 °C with 1 µM 5-HT (Sigma, St. Louis, MO), then fixed with 1% ultrapure formaldehyde, and diluted 1:20 in HEPES-Tyrode’s buffer. The samples were then permeabilized with 0.1% Triton X-100 and stained with Alexa Fluor 488-conjugated phalloidin (500 nM; Invitrogen Molecular Probes, Carlsbad, CA, USA) and, as a platelet identifier, phycoerythrin (PE)-conjugated CD41 (clone 5B12, DAKO, Carpinteria, CA, USA).

For determination of 5-HT-induced increases in platelet cytosolic calcium levels (assessed by Fluo-4 fluorescence), blood was diluted 1:10 in HEPES-Tyrode’s buffer and incubated for 30 min at 22 °C with 5 µM Fluo-4 (Invitrogen Molecular Probes), 1 mM probenecid (Invitrogen Molecular Probes; to reduce extracellular leakage of Fluo-4, as recommended by the manufacturer), and CD41–PE (as a platelet identifier). Samples were analyzed by flow cytometry for 30 s to establish the baseline Fluo-4 fluorescence in the absence of a platelet agonist, then 50 µL of 5-HT (10 µM final concentration, FC), ADP (20 µM FC) or TRAP (20 µM FC) was added and the samples were again monitored for at least 30 s to determine the new level of Fluo-4 fluorescence. Results are reported as the ratio of Fluo-4 fluorescence with/without agonist.

### Platelet surface CD63 antigen

Platelet surface CD63 antigen, also known as lysosomal membrane associated glycoprotein 3 (LAMP3), was detected using FITC-conjugated clone CLSGran/12 (catalog # IM1165U, Beckman Coulter, Fullerton, CA). Platelets were positively identified by staining with CD41a PerCP-Cy5.5 (BD Biosciences, San Diego, CA).

### Statistical analysis

Data analysis was performed using SAS (Version 9.2 or higher) or GraphPad Prism (Version 8.1 or higher). Descriptive statistics include mean ± standard deviation and median with interquartile range (IQR) for continuous variables. Categorical data were described as frequency with percentage and group comparisons performed with a Fisher exact test. A log transformation was used for the biomarkers with a right-skewed distribution. Where mean comparisons between SIDS and controls are presented, Student’s t-test was performed and where median comparisons are presented, a Wilcoxon rank sum test was performed. The Spearman correlation coefficient was used to assess the association between platelet and 5-HT biomarkers and post-conceptional age. An analysis of covariance (ANCOVA) modeling was used to assess the difference between SIDS and control groups, with adjustment for age and/or collection date. Correlation analyses were used to determine which covariates required adjustment. No prospective power calculation was possible since platelet biomarkers had not previously been measured in post-mortem blood samples.

### Supplementary Information

Supplementary Information.

## Data Availability

Data will be made available to researchers whose proposed use of the data has been approved. Please send requests for data sharing to Andrew.Frelinger@childrens.harvard.edu.

## Abbreviations

5-HIAA: 5-Hydroxyindole acetic acid
5-HT: 5-Hydroxytryptamine (serotonin)
ADP: Adenosine diphosphate
ANCOVA: Analysis of covariance
BZC: Benzoyl chloride
FITC: Fluorescein isothiocyanate
geoMFI: Geometric mean fluorescence intensity
IQR: Interquartile range
LC/MS: Liquid chromatography/mass spectrometry
LSmeans: Least squares means
GP: Glycoprotein
PE: Phycoerythrin
PerCP-Cy5.5: Peridinin chlorophyll protein-Cyanine5.5
SERT: Serotonin transporter
SIDS: Sudden infant death syndrome
TRAP: Thrombin receptor activating peptide
