# Supplementary material for: Dysregulation of platelet serotonin, 14–3–3, and GPIX in sudden infant death syndrome
Source: Sci Rep. 2024 May 15;14:11092. doi: 10.1038/s41598-024-61949-9 (PMC11096399; doi:10.1038/s41598-024-61949-9)
Supplement: Supplementary file 1 — Supplementary Information. [file 41598_2024_61949_MOESM1_ESM.pdf]

Supplemental Information
Dysregulation of Platelet Serotonin, 14-3-3, and GPIX in Sudden Infant Death Syndrome

Andrew L. Frelinger III, PhD[1]†*, Robin L. Haynes, PhD[2]†, Richard D. Goldstein, MD[3], Michelle A. Berny-Lang, PhD[1], Anja J. Gerrits, PhD[1], Molly Riehs[2], Elisabeth A. Haas, MPH[4], Brankica Paunovic, MD[5], Othon J. Mena, MD[6], Steven C. Campman, MD[5], Ginger L. Milne, PhD[7], Lynn A. Sleeper, ScD[8], Hannah C. Kinney, MD[2], Alan D. Michelson, MD[1]

†Contributed equally as co-first authors.

1. Center for Platelet Research Studies, Dana-Farber/Boston Children's Cancer and Blood Disorders Center, Harvard Medical School, Boston, MA, USA
2. Department of Pathology, Boston Children's Hospital and Harvard Medical School, Boston, MA, USA
3. Robert's Program on Sudden Unexpected Death in Pediatrics, Division of General Pediatrics, Division of General Pediatrics, Department of Pediatrics, Boston Children's Hospital and Harvard Medical School, Boston, USA
4. Rady Children's Hospital, San Diego, CA, USA
5. County of San Diego Medical Examiner's Office, San Diego, CA, USA
6. County of Ventura Medical Examiner's Office, Ventura, CA, USA
7. Division of Clinical Pharmacology, Vanderbilt University, Nashville, TN, USA
8. Department of Cardiology, Boston Children's Hospital and Department of Pediatrics, Harvard Medical School, Boston, MA, USA

***Corresponding Author:** Andrew L. Frelinger III, Ph.D., Director, Center for Platelet Research Studies, Dana-Farber/Boston Children's Cancer and Blood Disorders Center, Boston Children's Hospital, Karp 07212, 300 Longwood Avenue, Boston, Massachusetts, USA 02115-5737. Telephone: 617-919-2537. Fax: 617-730-4632. e-mail: Andrew.Frelinger@childrens.harvard.edu.

Supplemental Figures

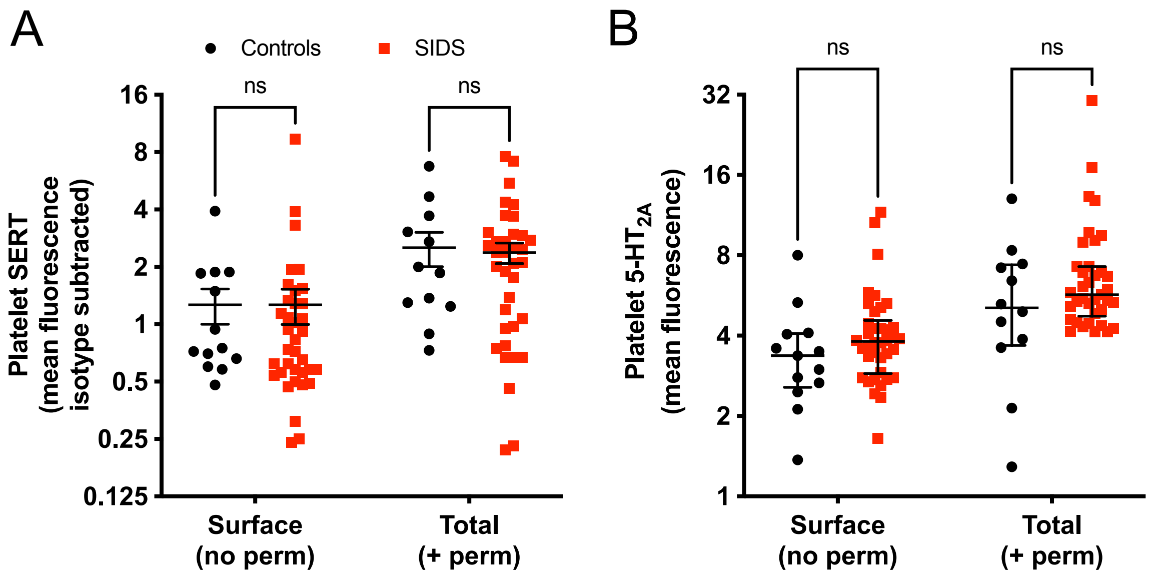

Figure S1. Platelet surface and total SERT and 5-HT2A in SIDS and Controls. Results are presented as mean fluorescence after subtraction of isotype-matched background. Data means ± SE for SIDS (n=36) and controls (n=12 – 13).

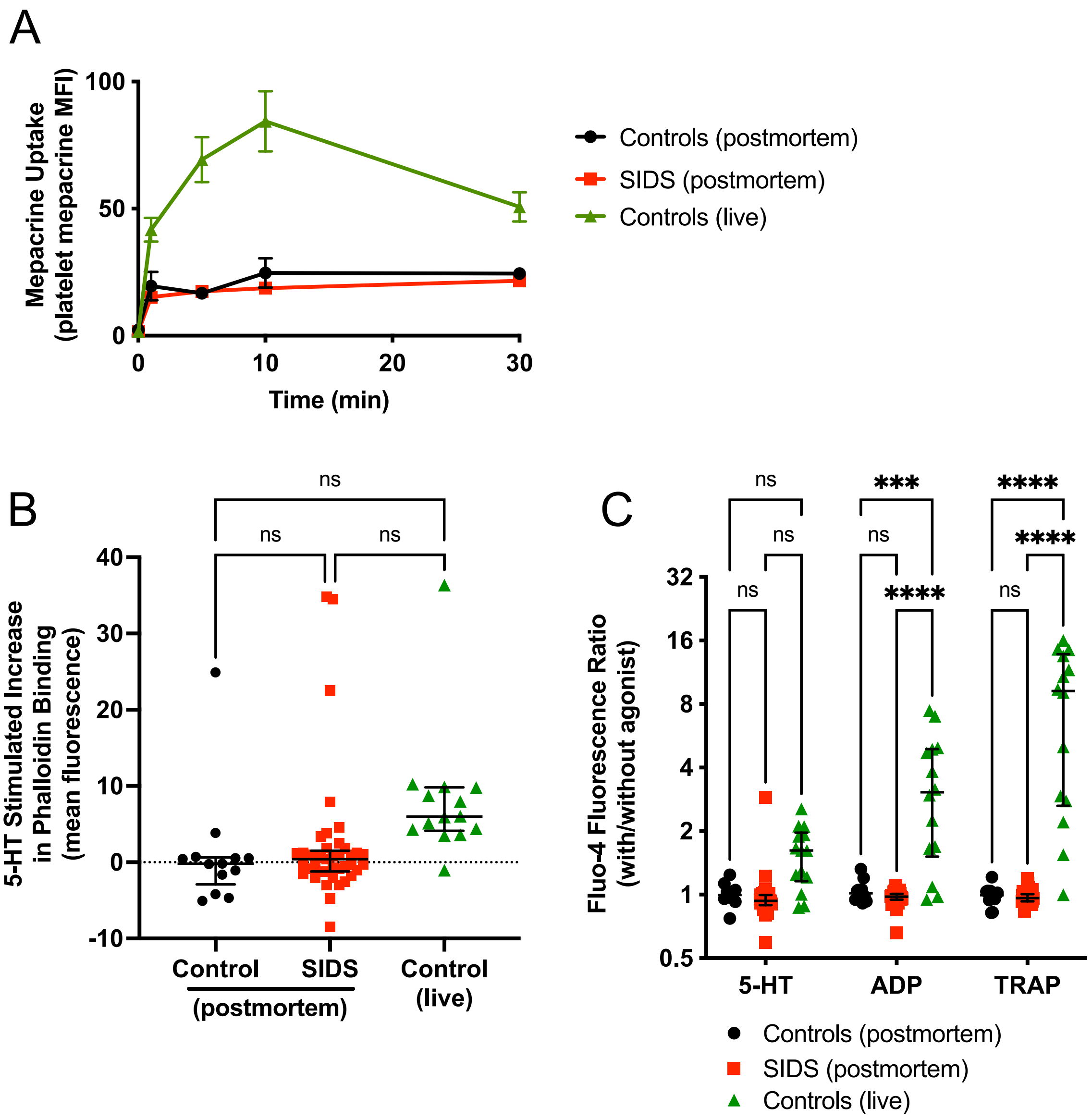

Fig. S2. SERT and 5-HT2A function are absent in platelets from aged postmortem blood, but not in platelets in freshly collected blood from living donors. (A) SERT function as measured by mepacrine uptake and 5-HT2A function as measured by (B) 5-HT-stimulated increase in phalloidin stained F-actin or (C) 5-HT-stimulated increase in cytosolic free calcium. Samples from living donors were tested within 4 hours of collection whereas SIDS and control samples were collected ~ 22 hours postmortem and tested more than 24 hrs later. Endpoints were measured by flow cytometry as described in the Supplementary Methods. Results are means ± SE for SIDS (n=41) and controls (n=12).

Supplemental Tables

Table S1. HPLC gradient for 5-HT, 5-HIAA, and histamine analysis.

Time	Flow rate	%A	%B	Gradient
0.00	0.200	99	1	--
0.10	0.200	93	7	6
0.50	0.200	85	15	6
14.0	0.200	45	55	6
14.5	0.200	30	70	6
18.0	0.200	1	99	6
19.0	0.200	1	99	6
19.1	0.200	99	1	6
22.0	0.200	99	1	6

Table S2. LC\MS settings for 5-HT, 5-HIAA, and histamine analysis.

	Retention Time	Precursor Ion	Product Ion	Collison Energy	Cone Voltage
BZC-5-HT	14.15	385	264	16	20
$^{13}C_6$-BZC-5-HT	14.15	397	270	16	20
BZC-5-HIAA	11.27	313	146	10	10
$^{13}C_6$-BZC-5-HIAA	11.27	319	146	10	10
BZC-Histamine	12.88	216	105	12	20
$^{13}C_6$-BZC-5-Histamine	12.88	222	111	12	20

Table S3. SERT and 5-HT_{2A} functional tests.

Biomarker Variable	SIDS (n=50) LSmean (SE)	Controls (n=13) LSmean (SE)	p-value
SERT: Mepacrine uptake			
MFI at 1 min	15.53 (1.79)	19.61 (3.60)	0.490[a,b]
MFI at 5 min	17.42 (1.17)	17.52 (2.36)	0.973[b]
MFI at 10 min	18.91 (1.94)	25.33 (3.90)	0.233[a,b]
MFI at 30 min	21.65 (1.58)	25.77 (3.17)	0.262[b]
5-HT_{2A}: Calcium flux, ratio of Fluo-4 fluorescence with/without 5-HT	0.98 (0.05)	1.00 (0.10)	0.711[a,b]
5-HT_{2A}: 5-HT stimulated increase in F-actin (change in phalloidin staining, MFI)	2.22 ± 8.64[c]	1.07 ± 7.58 [c]	0.670

[a]P-value for log-transformed data.
[b]Adjusted for post-conceptional age.
[c]LSmean ± SD
